# Characterization of a Novel Tanay Virus Isolated From *Anopheles sinensis* Mosquitoes in Yunnan, China

**DOI:** 10.3389/fmicb.2019.01963

**Published:** 2019-08-22

**Authors:** Lu Zhao, Caroline Mwaliko, Evans Atoni, Yujuan Wang, Yunzhi Zhang, Jianbo Zhan, Xiaomin Hu, Han Xia, Zhiming Yuan

**Affiliations:** ^1^Key Laboratory of Special Pathogens and Biosafety, Wuhan Institute of Virology, Chinese Academy of Sciences, Wuhan, China; ^2^University of Chinese Academy of Sciences, Beijing, China; ^3^Yunnan Institute of Endemic Disease Control and Prevention, Dali, China; ^4^Division for Viral Disease with Detection, Hubei Provincial Center for Disease Control and Prevention, Wuhan, China

**Keywords:** Tanay virus, *Negevirus*, insect specific viruses, yunnan, *Anopheles sinensis*

## Abstract

Globally, mosquitoes are known to be competent vectors to various arboviruses that cause serious and debilitating diseases to humans and animals. Conversely, mosquitoes harbor a wide array of insect specific viruses (ISVs) that are generally neglected. Extensive characterization of these ISVs is important in understanding their persistence infection effect on host behavior and arbovirus transmission. Herein, we report first time isolation of Tanay virus (TANAV) isolate YN15_103_01 in *Anopheles sinensis* mosquitoes from Yunnan Province, China. Phylogenetically, the isolate’s nucleotide identity had more than 14.47% variance compared to previous TANAV isolates, and it clustered into an independent branch within the genus *Sandewavirus* in the newly proposed taxon *Negevirus.* TANAV growth and high titers was attained in Aag2 cells (10^7^ PFU/mL) but with no CPE observed up to 7 days.p.i. compared to C6/36 cells that exhibited extensive CPE at 48 h.p.i. with titers of 10^7^ PFU/mL. Contrarywise, the viral isolate did not replicate in vertebrate cell lines. Electron microscopy analyses showed that its final maturation process takes place in the cell cytoplasm. Notably, the predicted viral proteins were verified to be corresponding to the obtained SDS-PAGE protein bands. Our findings advance forth new and vital knowledge important in understanding insect specific viruses, especially TANAV.

## Introduction

Arthropod-borne viruses (arboviruses) cause a significant part of the current global emerging and re-emerging infectious diseases, hence they pose a huge public health risk worldwide. The global spread, emergence and re-emergence of these arboviruses has been promoted by several factors including increased global trade (Cleton et al., 2012), diverse and changing environmental conditions (Soverow et al., 2009; Morin and Comrie, 2013) and the wide geographical distribution range of mosquitoes (Weaver and Reisen, 2010). Different arthropods including mosquitoes are known to spread arboviruses via complex replicative lifecycle in both the arthropod vector and vertebrate hosts (dual-host) (Vasilakis and Tesh, 2015; Halbach et al., 2017). Despite causing arbovirus infections, mosquitoes are also known to harbor a wide array of other viruses (Shi et al., 2016); commonly referred to as insect-specific viruses (ISVs) that persistently infect insects (single-host) and only replicate on insect cell lines. Their inability to infect mammalian hosts might be as a result of their heat sensitivity (Aliota et al., 2012; Marklewitz et al., 2015), lack of cell surface receptors and host factors (Nasar et al., 2015; Halbach et al., 2017; Hermanns et al., 2017). Because of their inability to cause diseases in humans and their far less or even absent economic impact on animals and plants, ISVs are largely neglected and very few have been identified and characterized in the past. However, modern-day advances in deep sequencing technology and bioinformatic tools have paved a way for the effective discovery of mosquito viruses. Consequently, many ISVs have been described from different mosquito species and majority of these newly discovered ISV’s phylogenetically cluster to similar viral groups associated with classical arboviruses. They include *Flavivirus* (Crabtree et al., 2003; Sang et al., 2003; Cook et al., 2006; Zuo et al., 2014; McLean et al., 2015), *Alphavirus* (Nasar et al., 2012), *Bunyavirus* (Marklewitz et al., 2011, 2013; Auguste et al., 2014), *Mesonivirus* (Vasilakis et al., 2014; Wang et al., 2017), *Almendravirus* (Contreras et al., 2017), *Reovirus* (Attoui et al., 2005; Hermanns et al., 2014; Auguste et al., 2015; Harrison et al., 2016), and *Negevirus* (Vasilakis et al., 2013; Auguste et al., 2014; Kallies et al., 2014; Nabeshima et al., 2014; Carapeta et al., 2015; Kawakami et al., 2016; Fujita et al., 2017; O’Brien et al., 2017; Moraes et al., 2019; Wang et al., 2019; Zhou et al., 2019). Moreover, they are thought to be the ancestors of arboviruses (Marklewitz et al., 2015). Possible application of these insect-specific viruses in vaccine development and use as biocontrol agents have been investigated (Bolling et al., 2015). It has been hypothesized that ISVs might act as natural regulators of arboviral infection, replication, and transmission (Hobson-Peters et al., 2013; Hall et al., 2016; Hall-Mendelin et al., 2016). Therefore, their high abundance, extensive viral taxa distribution and association to classical arboviruses warrants more in-depth research.

*Negevirus* is a new taxon of ISVs first described by Vasilakis’ team (Vasilakis et al., 2013). They are spherical or elliptical in shape with particle size of 45–55 nm in diameter. Their genomic RNA is non-segmented, polyadenylated positive sense strand with a genome length ranging from 9 to 10 kb. In addition, they have limited untranslated regions (UTRs) containing three major open reading frames (ORF’s). Some of the viruses in this taxon including Tanay virus (TANAV) (Nabeshima et al., 2014), Castlerea Virus (O’Brien et al., 2017) and Okushiri virus (Kawakami et al., 2016) have projection-like structures. This taxon has two groups, namely *Sandewavirus* and *Nelorpivirus*, which are distantly related to some plant viruses, such as *Cileviruses*, *Higrevirus*, and *Blunervirus* (Quito-Avila et al., 2013; Carapeta et al., 2015). Notably, negeviruses have been described to infect a wide range of hematophagous insects including 9 mosquito genera from the *Culicidae* Family: *Culex* spp., *Aedes* spp., *Anopheles* spp., *Armigeres* spp., *Psorophora* spp., *Uranotaenia* spp., *Deinocerites* spp., *Wyeomyia* spp. *and Trichoproson* spp. (Nunes et al., 2017). Geographically, negeviruses have been described in Asia (Vasilakis et al., 2013; Nabeshima et al., 2014; Hang et al., 2016; Kawakami et al., 2016; Fujita et al., 2017; Nunes et al., 2017; Wang et al., 2018; Zhao et al., 2018), Africa (Vasilakis et al., 2013; Kallies et al., 2014), Oceania (O’Brien et al., 2017), Europe (Carapeta et al., 2015), South America (Vasilakis et al., 2013; Nunes et al., 2017; Moraes et al., 2019), Central America (Auguste et al., 2014; Nunes et al., 2017), and North America (Vasilakis et al., 2013; Nunes et al., 2017; Charles et al., 2018), implying their wide global distribution. Downstream, experimental studies have demonstrated the ability of negeviruses to replicate and co-infect host cells with other viruses. For instance, a study conducted by Vasilakis et al. (2013) on adult *Ae. aegypti* and *Ae. albopictus* mosquitoes with Negev virus established that the virus was more likely transmitted via vertical or transovarial transmission rather than oral infection, relying on high viral titer among their insect hosts. In addition, Carapeta et al. (2015) reported that co-infection of Negevirus-OCNV virus forms an intermediate dsRNA, which could trigger a RNA silencing mechanism, by delivering dsRNA targeting the “lethal” gene transcript to the intended pathogen, this mechanism can knock down this gene and lead to insect mortality revealing that negeviruses hold great potential that can be applied in “species-specific” insect biological control strategies (Vogel et al., 2018). Thus, more information is required to understand the virus-host interaction in nature (Quito-Avila et al., 2013; Carapeta et al., 2015). Moreover, multiple hairpin structures have been predicted in both 5′ and 3′ UTRs, especially in the 5′-UTR region, there is an IRES (Internal Ribosome Entry Site) structure that allows for cap-dependent translation of the viral RNA (Gorchakov et al., 2014). Mutating the IRES element or adding an inhibitor, reduces the ability of the virus to replicate to produce attenuated strains (Pestova et al., 1996; Li et al., 2004). Therefore, negeviruses can be developed as a potential vehicle for vaccine production.

Tanay virus was first isolated in the Philippines from pools of *Culex* spp. and *Armigeres* spp., in 2013 (Nabeshima et al., 2014). In the same year, other strains of TANAV were isolated from *Culex tritaeniorhynchus* and *C. quinquefasciatus* mosquitoes in Guangxi, China (Wang et al., 2018). However, these two studies only described preliminarily findings on TANAV viral morphology, genome organization, and phylogeny. In the surveillance program of vector-borne viruses in mosquitoes in Yunnan, China, TANAV strain YN15_103_01 was isolated in our laboratory from *Anopheles sinensis*. Here, we describe its extensive characterization, including the viral structure, growth curve in mosquito and vertebrate cells, genome orientation, coding proteins and phylogeny. Our findings are important and crucial for a better understanding of TANAV properties and their effect on persistence infection in mosquitoes and arbovirustransmission.

## Materials and Methods

### Mosquito Collection and Sample Preparation

Adult *Anopheles sinensis* mosquitoes were trapped from Dehong, Lincang, Pu’er, Xishuangbanna and Honghe, Yunnan province, China, cities bordered Myanmar, Laos and Vietnam. Sampling was conducted between July and September, 2015. A total of 1722 adult mosquitoes were caught and assigned into 17 pools based on their respective collection sites. Each mosquito pool was triturated by Cryogenic grinding method at liquid nitrogen temperatures using sterile mortars and pestles. After sufficient grinding, 2 ml of Roswell Park Memorial Institute (RPMI) medium was added for homogenization (Wang et al., 2017). Mosquito macerates were clarified by centrifugation at 20,000 × *g* (4°C for 30 min) and filtered through a 0.22 μm membrane filter (Millipore, Billerica, United States) to remove cell debris and bacteria. Supernatants were stored at −80°C until further use.

### Cell Culture, Virus Isolation and Purification

Invertebrate cell lines (*Aedes albopictus* RNAi-deficient [C6/36], *Aedes aegypti* [Aag2]), as well as Vertebrate cell lines (African green monkey kidney [Vero], baby hamster kidney [BHK-21] and Human adrenal gland [SW13]) cell lines were used for this study. C6/36 and Aag2 cells were maintained at 28°C (in the absence of CO_2_) in RPMI Medium supplemented with 10% fetal bovine serum (FBS) and 1% penicillin/streptomycin. Vero, BHK-21 and SW13 cells were grown at 37°C in Dulbecco’s minimal essential medium (DMEM) (4.5 g/liter D-glucose) supplemented with 10% FBS and 1% penicillin/streptomycin.

The micro-filtered supernatants were used to inoculate sub-confluent C6/36 cell monolayers in 24-well plates. After 1 h of incubation at 28°C, the inoculum was removed and replaced with RPMI (2% FBS), and the culture plates incubated at 28°C for 7 days. Viral passaging was done in triplicates.

Virus purification was performed as previously described (Coelho et al., 2017; Xia et al., 2018). Briefly, in cultures where CPE was observed, the virus in the culture supernatant was harvested and clarified to remove cellular debris by Centrifugation at 5,000 × *g* (4°C for 30 min). Thereafter, ultracentrifugation was conducted by adding 2 ml of the harvested virus supernatant at the bottom of the ultracentrifuge tube and then followed by careful addition of 3 ml of 20% (wt/vol) sucrose in phosphate-buffered saline (PBS) buffer into tube. Ultracentrifugation was performed at 40,000 × *g* (4°C) for 2 h, in a Type 70 Ti rotor (Beckman). Upon completion, the supernatant was discarded. 200 μl of PBS was added to the sediment at the bottom of the tube and left to stand overnight at 4°C. Next, the precipitate was resuspended and loaded onto an ultracentrifuge tube containing a continuous gradient of 10 to 70% sucrose (wt/vol). Ultracentrifuge was performed at 38,000 rpm (4°C) for 4 h in a SW41 Ti rotor (Beckman). On completion, the formed virus fractions were carefully aspirated out while the sucrose solution was discarded. Virus purification step was then conducted through loading of the obtained virus fractions onto a new ultracentrifuge tube containing PBS and then centrifuged at 38,000 rpm (4°C) for 1 h. The purified virus was stored at −80°C.

### Morphological Characterization

Electron microscopy analyses was performed using negative contrast technique (Nabeshima et al., 2014; Wang et al., 2017). Formvar carbon-coated copper grid was incubated on the hanging drop of pure virosome for 10 min, stained with 2% phosphotungstic acid (PTA) and examined in Hitachi U8010 electron microscope (Japan) at 100 kV.

Ultrastructural analysis of ultrathin sections of infected C6/36 cells were consistent as previously described elsewhere (Guzman et al., 2018), using an FEI Tecnai G20 transmission electron microscope (FEI Company, United States) at 200 kV.

### Plaque Assay

Virus titrations were performed as described by Vasilakis’ team (Vasilakis et al., 2013). Virus progeny produced plaques on sub-confluent C6/36 cell monolayers in 24-well plates. The virus stocks were 10-fold serially diluted in cell culture growth medium. Thereafter, the wells were inoculated in duplicates with 0.1 ml of virus aliquots for 1 h, with gentle rocking every 15 min to prevent cell desiccation. Afterward, the virus inoculum was removed, and cell monolayers were overlaid with 750 μl of a medium consisting of a 1:1 mixture of 2% tragacanth suspension and 2 × RPMI with 5% FBS, 2% tryptose phosphate broth (TPB) solution, and 2% penicillin/streptomycin. Cells were incubated at 28°C in 5% CO_2_ for 3 days to allow plaque formation. The overlay was removed and monolayers were fixed with 750 μl of 10% formaldehyde in PBS for 30 min. The cells were then stained with 2% crystal violet in 30% methanol for 5 min at room temperature; excess stain was removed under running water, and plaques were counted and recorded as per the plaque forming assay (measured in PFU).

### Virus Replication Kinetics

Virus replication kinetic studies were undertaken to examine the host range of the virus. Two mosquito cell lines namely, C6/36 and Aag2, and three mammalian cell lines namely, Vero, BHK-21, and SW13, were plated in a 25-cm^2^ culture flask (2 × 10^6^ cells). C6/36 cells were inoculated with virus at MOIs of 1, 0.01, and 0.0001 PFU/cell, while Vero, BHK-21 and SW13 cells were inoculated with virus at MOIs of 1, 0.01 PFU/cell. Aag2 and C6/36 cells were inoculated with virus at MOIs of 0.001 PFU/cell, which was performed to compare the viral replication in two different mosquito cells. The cell-free supernatants of the infected cell lines were collected at periodic intervals, and each aliquot was tittered by plaque assay on C6/36 cells (Kuwata et al., 2013).

### Nucleic Acid Extraction and Genome Sequencing

To determine the full genome sequence of the viral isolate, total RNA was extracted using RNeasy mini kit (Qiagen, Germany) and strand-specific libraries were prepared using the TruSeq^®^ Stranded Total RNA Sample Preparation kit (Illumina, United States) following the manufacturer’s instructions. Sequencing was performed at Shanghai Biotechnology Corporation using the Illumina HiSeq 2, 500 (Illumina, United States) platform. Generated sequence reads were *de novo* assembled using Trinity in Galaxy platform (Göertz et al., 2019)^1^.

### Protein Analysis

The purified viral particles were lysed directly in a water-bath with 5 × SDS loading buffer [250 mM Tris-HCl, pH 6.8, 10% SDS (W/V), 0.5% bromophenol blue (W/V), 50% glycerol (W/V), 5% β-mercaptoethanol (W/V)] for 10 min at 100°C. Subsequently, sodium dodecyl sulphate polyacrylamide gel electrophoresis (SDS-PAGE) on a 10% polyacrylamide gel was employed to separate the viral protein bands, with the gel being stained with Fast Silver Stain using Protein Stains O (Sangon, China). Putative opening reading frame (ORF) organization was predicted by the Softberry program (Shahmuradov et al., 2017)^2^. Conserved domains were predicted by HHpred (Söding et al., 2005)^3^ and BLAST search on the National Center for Biotechnology Information (NCBI) database. Prediction of *trans-*membrane regions was performed using TMpred (Hofmann, 1993)^4^ and TMHMMv2.0 (Krogh et al., 2001)^5^. Visualization of transmembrane domains (TMDs) by Protter (Omasits et al., 2014)^6^.

### Phylogenetic Analysis

For identification and evolutional analyses, ORF1 amino acid (aa) sequences of the negeviruses and all members of the family *Virgaviridae* were aligned using Clustal Omega (Harte et al., 2004)^7^. Ambiguously aligned regions were removed and the methyltransferase, helicase and the RdRp conserved protein domains in ORF1 were connected without a gap (Kallies et al., 2014) by using BioEdit Sequence Alignment Editor. Phylogenetic trees were inferred in maximum likelihood/rapid bootstrapping run and 1000 bootstrap replications on XSEDE [RAxML-HPC2 on XSEDE (8.2.10)]^8^. The evolutionary tree was visualized and edited by Figtree and 1000 bootstrap replications.

## Results

### Virus Isolation, Morphology and Sequencing

**FIGURE 1:**
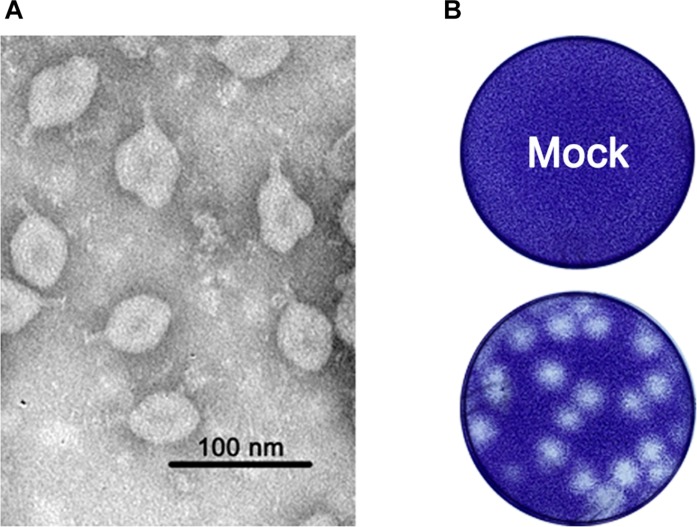
**(A)** Morphology of purified viral particles visualized by negative staining. **(B)** representative plaques of TANAV- isolate YN15_103_01 infected and uninfected (mock) C6/36 cells 48 h.p.i. Cells were fixed with 10% formalin and stained with crystal violet.

In the 17 homogenized samples of *Anopheles sinensis* mosquito pools, one caused exuberant CPE 48 h.p.i. after inoculation into the C6/36 cells, and the purified virion was one ellipsoid (about 40 × 60 nm) with a projection-like structure (16–20 nm) observed by the electron microscope (Figure 1A). Other than this, the viral particle was able to form large plaques with an unclear borderline in monolayers of C6/36 cells (Figure 1B). Thereafter, we acquired its full genome sequence through next generation sequencing. BLASTn analysis showed the sequence was closely related to TANAV isolate that was first reported from the Philippines (Nabeshima et al., 2014) and Guangxi, China (Wang et al., 2018). Hence, the viral isolate was named TANAV isolate YN15_103_01, since it was isolated from pool number #103. TANAV isolate YN15_103_01 complete genome sequence was assigned to GenBank under accession number MG673930, and the information of the sequence was published in the Genome announcement (Zhao et al., 2018).

### Virus Ultrastructural Characteristics

**FIGURE 2:**
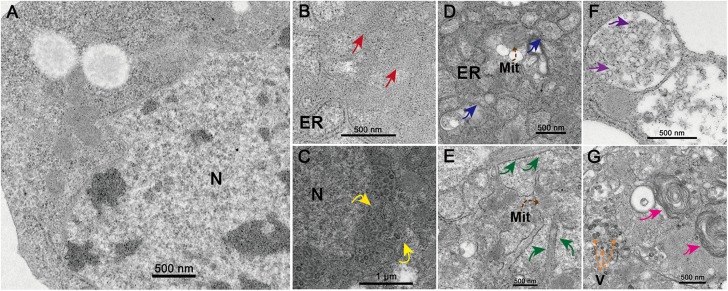
Transmission electron microscopy analysis of infected cells. Ultrastructure of mock-infected **(A)** and TANAV isolate YN15_103_01 infected (B-G) C6/36 cells at 48 h.p.i. **(B)** Para crystalline arrays could be observed surrounded by the ER (endoplasmic reticulum) (red arrows). **(C)** Expanded perinuclear space filled with vesicular (yellow arrows indicate the points of perinuclear membrane expansion). **(D)** Expanded ER stretch from the nuclear membrane to the cell membrane (blue arrows) and filled with microtubules **(E)** (brown dashed arrows), a few Mit (Mitochondria) were observed. **(F)** Cytoplasmic cytopathic vacuoles (CPVs), containing a lot of spherules (purple arrows) inside the vacuoles. **(G)** Several V (viroplasm-like particles) measuring approximately 40–50 × 60–70 nm (orange dashed arrows) and autolysosomes (pink arrows) were observed.

At 48 h.p.i. infected C6/36 cells exhibited clear and obvious CPE as most of them were rounded and ruptured (Supplementary Figure S1). Electron microscopy analysis revealed that the state of core organelles inside the C6/36 cells were enormously different in TANAV isolate YN15_103_01 infected C6/36 cells (Figures 2B–G), compared to the mock-uninfected cells (Figure 2A). The Perinuclear Space (PNS) of vesicles was dilated (Figure 2B), in line with previously described ultrastructural characteristic of negeviruses (Vasilakis et al., 2013; Auguste et al., 2014; Carapeta et al., 2015). In addition, the rough endoplasmic reticulum was deformed, expanding and extending from the nuclear membrane and to the cell membrane (Figures 2B–D) and filled with Para crystalline arrays (Figure 2B) and microtubules (Figure 2E). All of the above changes in organelles’ structures could be viral-exploited effects necessary for the proteins required for assembly of the virus, while the final virion maturation takes place in the cytoplasm (Figure 2G). Further, the formation of cytoplasmic cytopathic vacuoles (CPVs) (Figure 2F) and autolysosomes (Figure 2G) are the most prominent reasons for the observed CPE in the infected cells.

### Virus Replication

**FIGURE 3:**
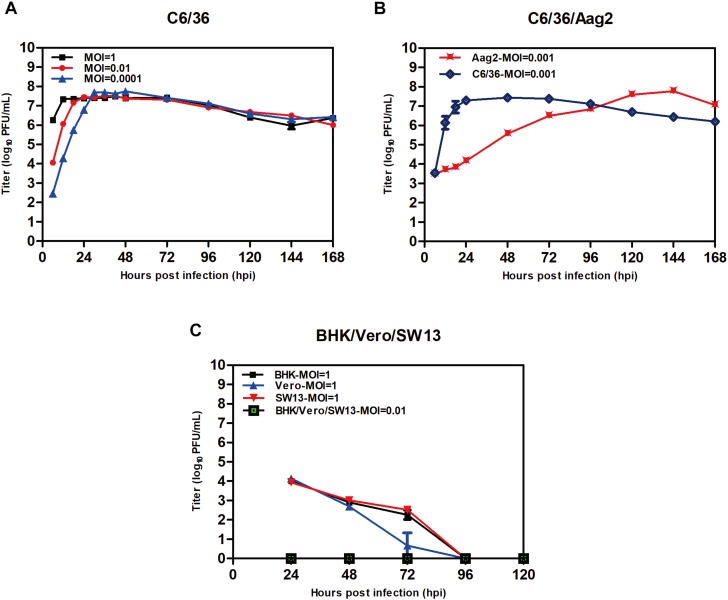
Replication of TANAV isolate YN15_103_01 in cells. **(A–C)** The growth curve of TANV isolate YN15_103_01 (MOI = 0.1–0.0001) was measured by Plaque assay at 6–168 h post infection.

TANAV isolate YN15_103_01-infected C6/36 cell lines produced extensive CPE at 24 h.p.i. 48 h.p.i. and 72 h.p.i. as MOI decreased from 1 to 0.01 and 0.0001 (Figure 3A). However, no overt cytopathic effects were observed in Aag2 cell line and three vertebrate cell lines up to 7 days post-infection p.i. (data not shown). The virus titers grown in the C6/36 cells reached maximal levels apace above 10^7^ PFU/mL at 12 (MOI = 1), 18 (MOI = 0.01) and 30 (MOI = 0.0001) h.p.i. Interestingly, despite the fact that TANAV isolate YN15_103_01 could grow to the highest titer in Aag2 similar to C6/36 when the MOI was 0.001, the growth is a slow rise in Aag2 compared to a sudden rise in C6/36 (Figure 3B). To determine the *in vitro* host range of TANAV isolate YN15_103_01, the cell-free supernatants of the infected vertebrate cell lines (Vero, BHK, SW13) with TANAV isolate YN15_103_01 were collected at 24, 48, 72, 96, and 120 h.p.i. and viral output was evaluated by the plaque assay on C6/36 cells. As mean replication titers remained steady (MOI = 0.01) or declined (MOI = 1), TANAV isolate YN15_103_01 failed to replicate in either of the vertebrate cell lines tested (Figure 3C).

### Virus Genome and Protein Analysis

**FIGURE 4:**
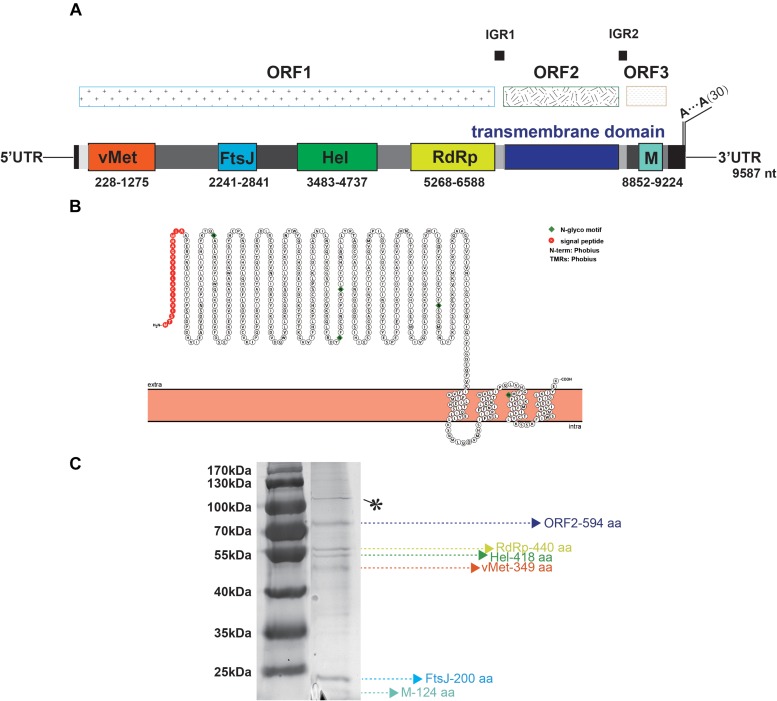
Bioinformatic and physical analysis of TANAV isolate YN15_103_01 structural proteins. **(A)** Three open reading frames (ORFs) were predicted in full genome, ORF1 encoded viral methyltransferase (vMet), RNA ribosomal methyltransferase (FtsJ), Helicase Helicase (Hel), RNA dependent RNA polymerase (RdRp) ORF2 contained transmembrane regions, ORF3 encoded membrane protein (M). **(B)** N-terminal signal peptide (S) and a C-terminal transmembrane domain (TM) of ORF2 predicted by protter. This protein contains 4 transmembrane (TM) regions. **(C)** Sodium dodecyl sulfate polyacrylamide gel electrophoresis (SDS- PAGE) analysis performed on purified virions shows 6 abundant proteins corresponding to the predicted ORF2 (594 aa), RdRp (440 aa), Hel (418 aa), vMet (349 aa), FtsJ (200 aa), M (124 aa), respectively, ^∗^ means no predicted functional protein was corresponding.

TANAV isolate YN15_103_01 is a 9, 587 nt single-stranded, positive sense virus with poly (A) tail. BLASTn analysis show it’s full genome shares a 79.02% nucleotide sequence identity to the TANAV that was isolated in Philippines (Nabeshima et al., 2014), and a 85.53% nucleotide sequence identity to the TANAV that was isolated from Guangxi, China (Wang et al., 2018). There are three Putative open reading frames (ORFs); ORF1, ORF2 and ORF3. They are flanked by UTRs, while each ORF is separated by two short intergenic regions. ORF1 starts at nt 78 to 6725, and it contains four putative functional protein domains: (i) a viral methyltransferase (vMet) at nt 228 to 1275; (ii) an RNA ribosomal methyltransferase (FtsJ) at nt 2241 to 2841; (iii) a Helicase (Hel) at nt 3483 to 4737; and (iv) an RNA-dependent RNA polymerase domain (RdRp) at nt 5268 to 6588. ORF2 starts at nt 6749 to 8533 and is predicted to contain four transmembrane regions with 20 aa N-terminal signal peptide, according to the protter software (Figures 4A,B). This result is consistent with the other reported strains of TANAV (data not shown). ORF3 ranges from 8663–9310 nt, which edited putative virion membrane protein (Figure 4A). To further analyze the major structural proteins of TANAV, proteins bands were observed. Six of the bands were corresponding to the predicted ORF2 (594 aa), RdRp (440 aa), Hel (418 aa), vMet (349 aa), FtsJ (200 aa), M (124 aa), respectively. Further, we also found an approximately 100-kDa protein from virus. However, its size is not consistent with the prediction from the full-length ORF sequences (marked as^∗^) (Figure 4C). The genomic RNAs included three open reading frames ORF1, ORF2, and ORF3, and the amino acid sequence similarity among the strains of TANAV was 89.75–95.12%, 73.55–88.22%, and 92.09–96.28%, respectively.

### Phylogenetic Analysis

**FIGURE 5:**
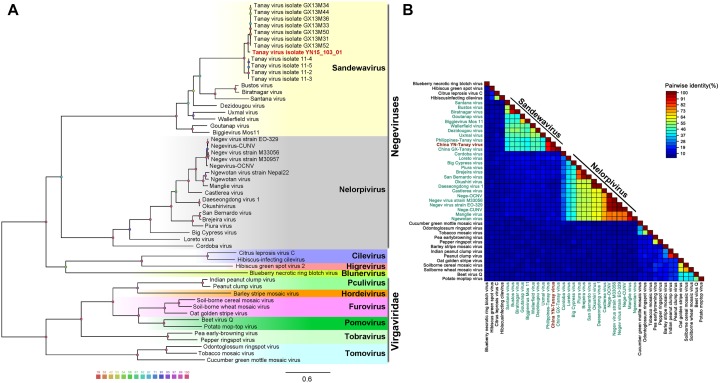
Analysis of a gap-free concatenated alignment of fused methyltransferase, viral helicase and RdRp domains of members of negeviruses, cileviruses, higreviruses and blunerviruses, as well as representative members of each genus of the Virgaviridae family. **A:** ML-Tree; **B:** SDT: TANAV isolate YN15_103_01 (red font); negeviruses (green font). Data shown on Supplementary File S1.

Unrooted gap-free phylogenetic trees were constructed based on concatenated amino acid sequences of fused methyl transferase, viral helicase and RdRp domains. The results indicate that TANAV strains could be clearly divided into two small groups; TANAV isolate YN15_103_01 laid on a small independent branch but closer to Guangxi strains than from the one from Philippines. All of them belong to the subclade *Sandewavirus* in *Negevirus* (Figure 5A). Following discovery of more *Negevirus* species, the two clades tentatively named *Nelorpivirus* and *Sandewavirus* are more likely to be proven to form taxonomic groups on genus level from their respective evolutionary relationships and phylogenetic distances (Figure 5B).

Other than this, the topology tree of *Negevirus* with plant-infecting viruses of the genera *Cilevirus*, *Higrevirus* and *Blunervirus* was debatable. Here, we found the out-group viral family *Virgaviridae* formed a solitary branch, and genera *Cilevirus*, *Higrevirus*, and *Blinervirus*as the closest but still distant relatives formed the other branch with *Negevirus*, which was more closely genetically related to *Nelorpivirus* than *Sandewavirus* in previous reports (Nunes et al., 2017). However, the topology that *Nelorpivirus* was more closely to *Sandewavirus* than genera *Cilevirus*, *Higrevirus*, and *Blinervirus* was more likely in the light of similar genome organizations and hosts cluster (Kallies et al., 2014).

## Discussion

In this study, we isolated and characterized Tanay virus-isolate YN15_103_01, from *Anopheles sinensis* mosquitoes collected in Yunnan province, China. Our isolate’s nucleotide identity was 79.02% similar to the TANAV that was reported in Philippines (Nabeshima et al., 2014), and 85.53% identical to the TANAV reported from Guangxi, China (Wang et al., 2018). TANAV phylogenetically clusters under genus *Negevirus* (Figure 5): a new taxon that contains insect specific viruses (ISVs) including Negev virus, Goutanap virus, Bustos virus, Santana virus, Uxmal virus and Manglia virus, among others. Morphologically, the viral particle of TANAV is distinctive with a projection-like structure (Figure 1A). This finding is in line with previously conducted studies (Nabeshima et al., 2014). However, we did not identify the projection-like structure of the virus particles in the cell sections and know when it was assembled. This can be due to: (1) we did not choose good timing and angle of the slice or; (2) perhaps the tail is packaged when it is budding from the virus or it was a; (3) limitation of the used microscope. The recently developed cryo-EM, that has less artifact-prone alternative to thin-section, might be the best instrument of observation in this case (Serwer et al., 2018). Besides, the TANAV virus projection-like structure is comparable to the injection needle of phages (Guan et al., 2019), hence future studies should investigate whether the structure plays a role in virus binding to the host cell receptors, increasing its adsorption during virus entry. Such studies may employ use of reverse genetics to generate a mutant viral strain that lacks the projection-like structure.

Tanay virus did not replicate in the vertebrate cell lines tested, implying it is not an infectious agent in vertebrates. Steady TANAV growth and high titers were observed in Aag2 cells but with no CPE observed up to 7 days pi. compared to C6/36 cells that exhibited extensive CPE after 48 h.p.i. The non-cytopathic growth of TANAV in Aag2 cells might be due to establishment of a latent infection by the virus in *Ae. aegypti* mosquitoes. Unlike C6/36 cell lines that are Dicer-2 deficient, Aag2 cell lines are able to produce viral siRNAs, in turn the siRNA response suppresses viral replication processes in the *Ae. aegypti* cells to low tolerant levels in the host cell, thus supporting a lasting infection with no or minimal cytopathic effects (Göertz et al., 2019). Hence, *Ae. aegypti* mosquitoes might be natural reservoir hosts of TANAV in natural environment habitat. Further, TANAV may be infecting several mosquito species in nature since past reported studies, including ours, have described isolation of TANAV virus from *Culex, Armigeres* and *Anopheles* mosquitoes. However, recently described Uxmal virus was found not to replicate in *Anopheles* mosquito cells, suggesting its insect host range is restricted (Charles et al., 2018). Therefore, it is essential to study the host range, virulence, and tissue tropism of negeviruses across different mosquito species. This information will help to elucidate how negeviruses (including TANAV) are maintained in nature. In addition, *in vivo* and *in vitro* co-infection of TANAV with other representative strains of classical arboviruses should be carried out in mosquitoes and mosquito cell lines to detect whether it has any effect on infectivity and virulence of these arboviruses.

The TANAV genome architecture comprised of three putative open reading frames (ORFs): ORF1, ORF2 and ORF3 located at positions 78 to 6725 nt, 6749 to 8533 nt and 8, 663–9, 310 nt, respectively. The hypothetical ORF3 of negeviruses share a conserved sequence similar to the p24 protein of CiLV-C (YP_654543) (Nabeshima et al., 2014). Specifically, ORF1 comprised of four putative functional protein domains namely viral methyltransferase (vMet), RNA ribosomal methyltransferase (FtsJ), Helicase (Hel) and RNA-dependent RNA polymerase domain (RdRp) (Figure 4). ORF1 protein Helicase function contained a conserved tobacco mosaic virus replicase subunit alpha/beta domain (3VKW_A). Although little is known about the associations between negeviruses and plant viruses, these observations raise the possibility of cross-kingdom virus transfer between insects and plants in ancient times. In further characterization of Endogenous Viral Element (EVE) candidates derived from virga/nege-like viruses in insect genomes, within a clade of the insect tobacco-like group (containing a conserved tobacco mosaic virus (TMV)-coat superfamily domain (accession CL20208), obtained data strongly supports the view that insect EVE candidates related to virga/nege-like viruses may be the footprints of ancient insect RNA viruses, but not plant RNA viruses (Kondo et al., 2019), and might indicate there would be a larger taxonomic group including plant viruses and negeviruses. Hence, we hypothesize that if negeviruses evolved from plant viruses, then they ought to have undergone a two-host adaptation phase. Therefore, there must be some viruses that can replicate in plants and insect cells. To ascertain this hypothesis, studies that test growth of negeviruses on plant cells and/or plant viruses (like cileviruses, higreviruses and blunerviruses) growth on insect cells should be designed and carried out.

**FIGURE 6:**
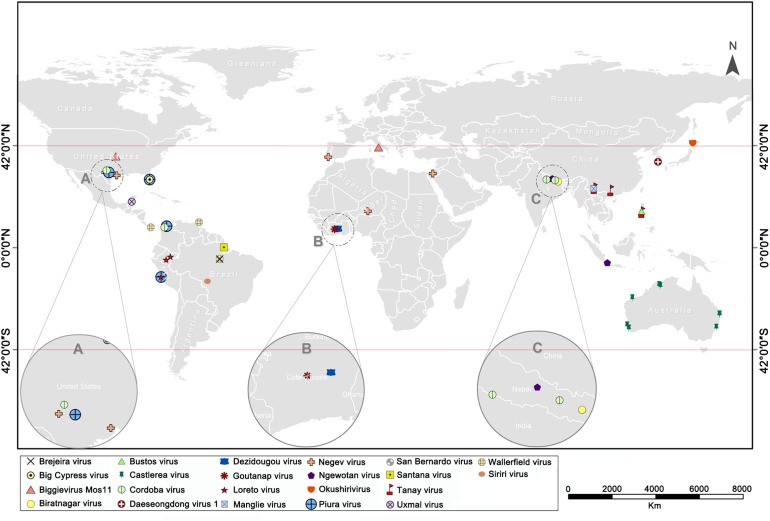
World map showing the global distribution of negeviruses. Data shown on Supplementary File S1. **(A–C)** indicates a partially enlarged area and numbers and sorts them from left to right.

The continuous rise in discovery of ISVs, including negeviruses, has presented a promising and exciting field of study in mosquito-virus biology. So far, about 22 negevirus species have been described worldwide (Figure 6). These negeviruses appear to be limited to latitudes 42°N and 42°S; which falls on tropical and sub-tropical temperate regions, indicating that their distribution could be influenced significantly by environmental and climatic factors. Therefore, new insights on negeviruses and related ISVs, including TANAV, will offer far reaching important and profound knowledge not only to the biological impact on mosquito populations but also to ongoing translational studies that aim to manipulate mosquito specific viruses as biological agents and vaccines against classical arboviruses that are more common in temperate regions, worldwide.

## Data Availability

The datasets generated for this study can be found in the TANAV isolate YN15_103_01 complete genome sequence was assigned to GenBank under accession number MG673930.

## Author Contributions

ZY, HX, and LZ designed the experiments. LZ and YW performed the experiments. LZ, CM, and HX analyzed the data. ZY, HX, YZ, and JZ contributed reagents, materials, and analysis tools. LZ, CM, EA, XH, HX, and ZY wrote the manuscript.

## Conflict of Interest Statement

The authors declare that the research was conducted in the absence of any commercial or financial relationships that could be construed as a potential conflict of interest.

## Footnotes

1 http://mississippi.snv.jussieu.fr

2 http://www.softberry.com/berry.phtml?topic=virus0&group=programs&subgroup=gfindhttp://linux

3 https://toolkit.tuebingen.mpg.de/tools/hhpred

4 https://embnet.vital-it.ch/software/TMPRED_form.html

5 http://www.cbs.dtu.dk/services/TMHMM/

6 http://wlab.ethz.ch/protter/start/

7 http://www.ebi.ac.uk/services

8 https://www.phylo.org/portal2/tools.action

## Supplementary Material

FIGURE S1 Replication of TANAV isolate YN15_103_01 in C6/36 mosquito cells. Phase contrast photomicrographs of infected cells at 24–96 h.p.i. (MOI = 1, 0.01, 0.0001) and mock-infected (Objective lens × 10).

FILE S1 Detailed information of data used in making Figure 5 (all marked in red) on “Negeviruses phylogenetic tree” and Figure 6 (all isolated Negeviruses) on “Global distribution of Negeviruses”.
