# Supplementary material for: Characterization of a Novel Tanay Virus Isolated From Anopheles sinensis Mosquitoes in Yunnan, China
Source: Front Microbiol. 2019 Aug 22;10:1963. doi: 10.3389/fmicb.2019.01963 (PMC6714596; doi:10.3389/fmicb.2019.01963)
Supplement: FIGURE S1 — Replication of TANAV isolate YN15_103_01 in C6/36 mosquito cells. Phase contrast photomicrographs of infected cells at 24–96 h.p.i. (MOI = 1, 0.01, 0.0001) and mock-infected (Objective lens × 10). [file Data_Sheet_1.docx]

**SUPPLEMENTARY FIGURE**

**
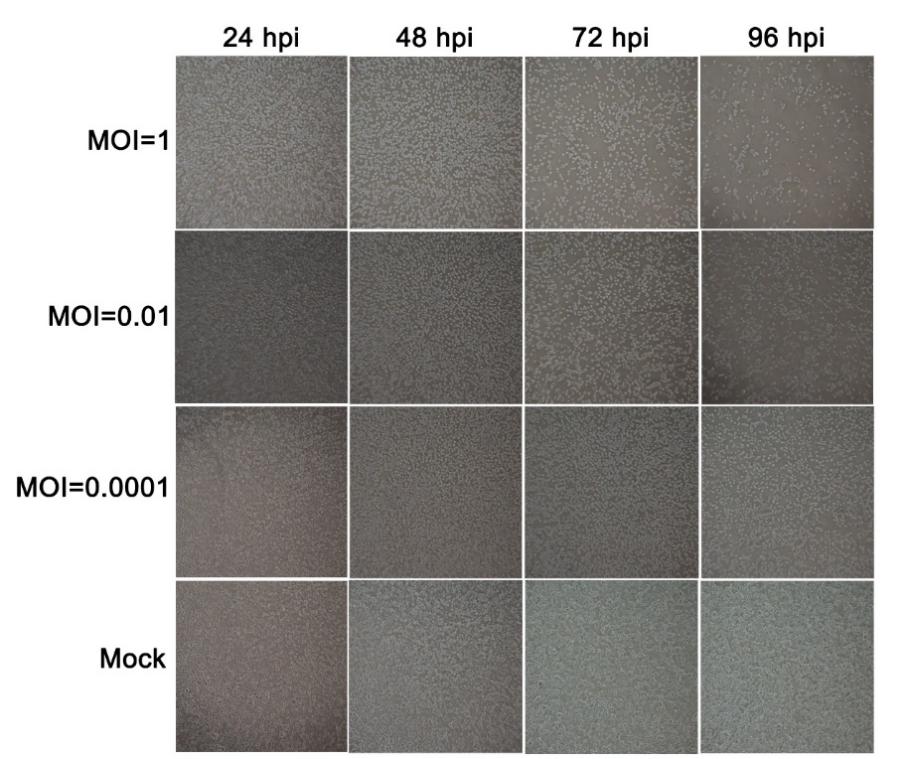
**

Supplementary Figure 1: Replication of TANAV isolate YN15_103_01 in C6/36 mosquito cells. Phase contrast photomicrographs of infected cells at 24-96 h p.i. (MOI=1, 0.01, 0.0001) and mock-infected (Objective lens×10).
